# Translational Control in Stem Cells

**DOI:** 10.3389/fgene.2018.00709

**Published:** 2019-01-15

**Authors:** Soroush Tahmasebi, Mehdi Amiri, Nahum Sonenberg

**Affiliations:** ^1^Department of Pharmacology, University of Illinois at Chicago, Chicago, IL, United States; ^2^Goodman Cancer Research Center, McGill University, Montreal, QC, Canada; ^3^Department of Biochemistry, McGill University, Montreal, QC, Canada

**Keywords:** translational control, stem cell, protein synthesis, development, mRNA

## Abstract

Simultaneous measurements of mRNA and protein abundance and turnover in mammalian cells, have revealed that a significant portion of the cellular proteome is controlled by mRNA translation. Recent studies have demonstrated that both embryonic and somatic stem cells are dependent on low translation rates to maintain an undifferentiated state. Conversely, differentiation requires increased protein synthesis and failure to do so prevents differentiation. Notably, the low translation in stem cell populations is independent of the cell cycle, indicating that stem cells use unique strategies to decouple these fundamental cellular processes. In this chapter, we discuss different mechanisms used by stem cells to control translation, as well as the developmental consequences of translational deregulation.

## Introduction

### The Importance of Translation Control in Mammalian Cells and Stem Cells

The abundance of proteins in a mammalian cell varies by several orders of magnitude (10^3^–10^8^ molecules per cell) (Li et al., 2014; Li and Biggin, 2015). Transcription rate, messenger RNA (mRNA) turnover, translation rate, and protein degradation are four fundamental cellular processes that regulate protein abundance. The poor correlation between protein and mRNA abundance, which is documented in numerous studies, and higher conservation of protein expression compared to mRNA expression across species suggest that post-transcriptional control explains a large percentage of protein variability (Gygi et al., 1999; Maier et al., 2009; Vogel et al., 2010; Schwanhausser et al., 2011; Aviner et al., 2013; Khan et al., 2013; Sharma et al., 2015). Parallel measurements of mRNA and protein levels along with mRNA and protein turnover demonstrated that the translation rate plays a dominant role in regulating the cellular proteome (Schwanhausser et al., 2011). Others reported a much higher correlation between mRNA and protein levels (*R*^2^ ≅ 0.6–0.9) (Li et al., 2014; Jovanovic et al., 2015). Nevertheless, these studies suggest that cell status dictates the contribution of transcriptional versus translational control in defining the proteome of the cell. During steady state or after long-term differentiation/adaptation, transcriptional control is considered the main determinant of the cellular proteome, whereas during early stages of state transition (differentiation/adaptation), translational control plays a dominant role (Lu et al., 2009; Ingolia et al., 2011; Kristensen et al., 2013). Translational control allows cells to quickly respond to internal and external stimuli before a new transcription program comes into effect (Liu et al., 2016).

Notably, among different proteins in the cells the levels of transcription factors and proteins performing essential cellular processes (e.g., ribosomal and mitochondrial proteins), are more stringently subjected to translational control (Lee et al., 2013; Jovanovic et al., 2015). This exquisite dependency on translational control, also referred to as “translation on demand,” has been well documented during early developmental stages, a period when transcription is known to be silenced. For instance, selective translational upregulation of few transcription factors (e.g., *Nanog*, *Sox19b*, and *Pou5f1*) is essential for activation of zygotic genome and maternal-to-zygotic transition (MZT) in zebrafish (Lee et al., 2013). The regulatory information encrypted in the 5′ and 3′ mRNA untranslated regions’ (UTRs) sequences plays a critical role in rendering a subset of mRNAs sensitive to translational control (Hinnebusch et al., 2016). Ribosome footprinting analysis underscored the importance of the upstream open reading frame (uORF) in translational control of several key pluripotency factors, such as *Myc* and *Nanog* (Ingolia et al., 2011). In addition to the importance of translational control in defining the cellular proteome, translational control also impacts transcription. A recent study uncovered a delicate fine-tuning between translation and transcription in embryonic stem cells (ESCs) and peri-implantation embryos. An acute inhibition of global translation (using cycloheximide or mTOR inhibitors) disrupts the hypertranscription and euchromatic state of ESCs (Bulut-Karslioglu et al., 2018). This finding highlights the importance of coordination between transcription and translation for maintenance of self-renewal and pluripotency.

### Initiation, the Rate Limiting Step of Translation

mRNA translationis divided into four steps; initiation, elongation, termination, and ribosome recycling. Initiation is the process through which the small subunit of the ribosome (40S), as a component of the 43S preinitiation complex, is recruited to the mRNA, and scans the mRNA 5′UTR from 5′ to 3′ to recognize the start codon. Following recognition, the 80S initiation complex is assembled at the start codon and elongation will proceed (Sonenberg and Hinnebusch, 2009; Hinnebusch et al., 2016).

Eukaryotic ribosomes (consisting of 4 ribosomal RNAs and 80 ribosomal proteins) are not fully equipped to directly bind to mRNAs and hence, start translation. The activities of multiple eukaryotic translation initiation factors (eIFs) are therefore required for recruitment of ribosomes to mRNAs and translation initiation. The orchestrated activity of eIFs culminates in the assembly of two multisubunit complexes, the 43S preinitiation complex (consist of small ribosomal subunit, initiator tRNA, and eIF1, 1A, 2, and 3) and the eIF4F complex (consist of eIF4E, eIF4A, and eIF4G) at 5′ end of mRNA. In eukaryotic cells, the abundance of a key component of the eIF4F complex, cap-binding protein eukaryotic translation initiation factor 4E (eIF4E), is far less than that of ribosomes [41 × 10^4^ molecules of eIF4E compared to 1064 × 10^4^ cytosolic ribosomes per HeLa cell (Merrick and Pavitt, 2018)], which makes eIF4E availability the limiting factor for translation initiation. The activity of eIF2B has been also identified as a rate-limiting step in translation initiation. The eIF2B is a guanine nucleotide-exchange factor (GEF) that converts eIF2.GDP to eIF2.GTP, a critical step requires for the formation of the 43S preinitiation complex. Consequently, most mammalian cells, including stem cells, have a surplus of non-translating ribosomes, which could be engaged in translation through the control of the activity of eIFs. Several signaling pathways such as the mechanistic Target of Rapamycin (mTOR), the mitogen activated protein kinase (MAPK), and the integrated stress response (ISR) control translation through phosphorylation of activators (e.g., eIF4E and eIF2α) or inhibitors [e.g., 4E-BPs (eIF4E-binding proteins; inhibitors of eIF4E), PDCD4 (Programmed Cell Death 4; an inhibitor of eIF4A)] of translation initiation. This provides a tunable translation regulatory system that adjusts the translation rate, according to cellular demands.

## Global Translation is Inhibited in Stem Cell Populations

**FIGURE 1:**
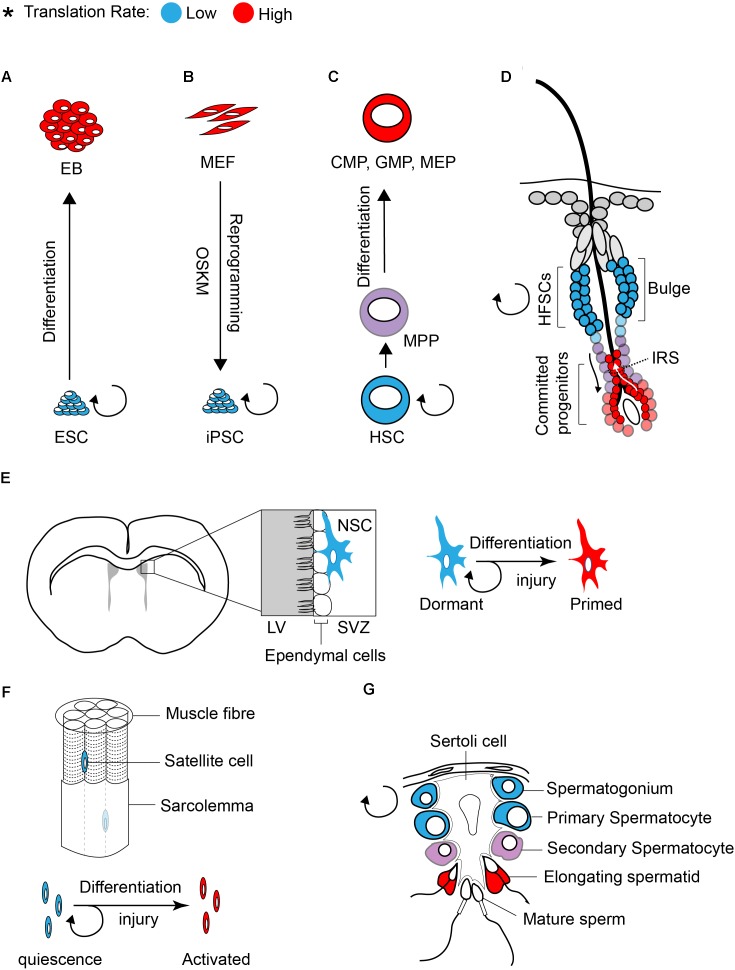
Translation inhibition is a hallmark of stem cells. The rate of protein synthesis in pluripotent ESCs **(A)** or iPSCs **(B)** and in multipotent adult stem cells **(C–G)** is lower compared to early differentiating cells or progenitors. Blue and red color defines low and high translation rates, respectively.

Studies in both embryonic and adult stem cells demonstrated that stem cells require low translation rates to maintain an undifferentiated status (Figure 1; Sampath et al., 2008; Signer et al., 2014; Blanco et al., 2016; Zismanov et al., 2016). Sampath et al. (2008) first found that global translation is low in undifferentiated ESCs compared to EB (embryoid body). Differentiation [5 days culture in the absence of LIF (leukemia inhibitory factor)] increases polysome density in the differentiating cells by ∼60% and [^35^S] methionine incorporation by ∼2-fold as compared to undifferentiated ESCs. The increase in translation of differentiated cells coincides with a significant increase in the content of total RNA (∼50%), ribosomal RNA (∼20%), and proteins (∼30%).

Similar to ESCs, global translation is suppressed in somatic stem cells. Studies on various tissue specific stem cells such as hematopoietic stem cells (HSCs), hair follicle stem cells (HFSCs), and muscle stem cells (satellite cells) demonstrated that protein synthesis is restricted in stem cell population and is increased upon differentiation (Signer et al., 2014; Blanco et al., 2016; Zismanov et al., 2016).

Tight control of translation is crucial for the maintenance of HSCs, as only a 30% decrease (using *Rpl24^Bst/+^* mice, where ribosome protein Rpl24 is partially depleted) or increase (using *Mx1-Cre; Pten^fl/fl^* mice, where *Pten* is depleted from adult hematopoietic cells) in protein synthesis is sufficient to impair the proliferation and self-renewal of HSCs (Signer et al., 2014). The rate of protein synthesis also impacts normal hair cycle through regulation of the self-renewal and differentiation of HFSCs (Blanco et al., 2016). Activation of HFSCs during hair growth (transition from telogen to anagen) coincides with a profound increase in protein synthesis (Figure 1D). Committed progenitor cells located at the inner root sheath (IRS) display the highest translation rate compared to other progenitors. The importance of translation control in regulating HFSC has been highlighted in NOP2/Sun RNA Methyltransferase Family Member 2 (NSUN2) knockout (KO) mouse (Blanco et al., 2016). NSUN2 is an RNA methyltransferase that converts cytosine to 5-methylcytosine (m5C), and is required for decoding activity and stability of tRNAs. Hypomethylated tRNAs that are accumulated in NSUN2 KO cells, are cleaved by endonuclease and the resulting tRNA fragments inhibit translation initiation (Spriggs et al., 2010; Ivanov et al., 2011; Sobala and Hutvagner, 2013). NSUN2 is highly expressed in committed progenitor cells of the epidermis. The inhibition of translation in NSUN2 KO cells blocks the differentiation of epidermal stem cells toward committed progenitors, which leads to cyclic alopecia in the mouse (Blanco et al., 2016).

Lack of Pseudouridylate Synthase 7 (PUS7) has the opposite effect to that of NSUN2 deficiency. PUS7 is a member of pseudouridine synthases (PUSs) that catalyzes the pseudouridylation (Ψ) of a subset of tRNAs at U8 (uridine at position 8 of tRNA). Pseudouridylation of a group of tRNA-derived small fragments inhibit translation initiation, and consequently, the absence of PUS7 promotes global translation. Interestingly, a recent study uncovered the importance of PUS7 activity in maintenance and differentiation of ESCs and HSCs (Guzzi et al., 2018).

Translational control also plays a central role in differentiation of adult stem cells in the testis. This has been well documented in several translationally defective mouse models including in NSUN2 KO mouse. In addition to the epidermis, NSUN2 is highly expressed in testis and plays a critical role in germ cell differentiation. Consequently, NSUN2 KO males not only have defect in hair growth but they also display infertility. During the late stages of spermatogenesis, translation activation of germ cell-specific mRNAs is required for successful generation of spermatozoa (Figure 1G). Inhibition of global translation due to NSUN2 depletion halts the progression of a germ cell through the late stages of spermatogenesis, engendering infertility. Interestingly, a similar phenotype (male infertility and defect in late spermatogenesis) has also been reported in Paip2a {Pabp [poly(A)-binding protein]-interacting protein 2A} KO mice, where global translation is inhibited (Yanagiya et al., 2010). Three Pabp – interacting proteins (Paips) have been discovered in mammals [Paip1 (Craig et al., 1998), Paip2a (Khaleghpour et al., 2001), and Paip2b (Berlanga et al., 2006)]. This family of proteins regulates mRNA translation and stability through the control of PABP function. Lack of PAIP2s has been linked to translation activation as their bindings to PABP compete with the interaction of PABP with the poly(A) tail and eIF4G (Khaleghpour et al., 2001; Karim et al., 2006). During late spermatogenesis, translational derepression of a subset of mRNAs, such as Prms (protamines) and Tps (transition proteins), is essential for the generation of functional spermatozoa. This translational derepression coincides with shortening of poly(A) tails, from approximately 180 nucleotides in a translationally repressed state to 30 nucleotides in a translationally active state (Kleene, 1989). Conversely, lack of PAIPs during spermatogenesis inhibits translation of Prms and Tps. This effect has been explained by an excess expression of Pabpc1 (an isoform of Pabp that is expressed in Elongating spermatids) (Yanagiya et al., 2010). Altogether, these findings demonstrate that translational control is a key modulator of stem cell differentiation.

## How Do Stem Cells Maintain a Low Translation Rate?

### Ribosome Biogenesis

**FIGURE 2:**
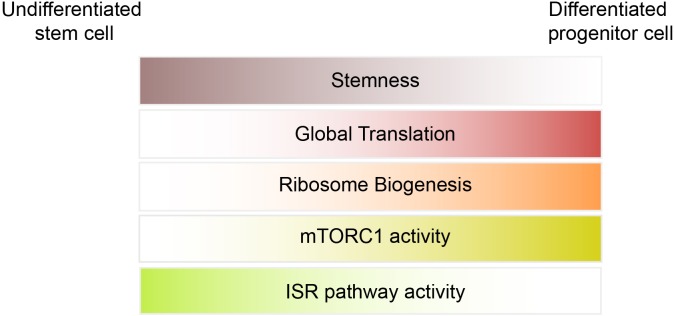
Underlying mechanisms of translation inhibition in stem cells.

Under physiological condition, ribosome abundance is not considered a limiting factor for translation initiation in stem cells (Figure 2). However, studies in *Drosophila* and mammals suggest that differentiation of stem cells relies on increased ribosomal biogenesis (Ingolia et al., 2011; Zhang et al., 2014; Sanchez et al., 2016). Sampath et al. (2008) found that ribosomal RNAs are ∼20% elevated in 5 day EB as compared to mESCs. Using ribosome footprinting, Ingolia et al. (2011) identified a modest increase in translation of ribosomal proteins (RPs) mRNAs at early stages of differentiation (36 h after LIF withdrawal), whereas translation of RPs strongly suppressed at later time points (8 days EBs). They concluded that the increase in expression of RPs at early stages of differentiation is required for the profound increase in global translation observed at later stages and is mediated by mTORC1 activation.

Single cell sequencing of neural stem cells (NSCs) demonstrated that in response to injury, there is a dramatic increase in transcription of the genes involved in ribosome biogenesis (Llorens-Bobadilla et al., 2015). The increase in ribosome biogenesis triggers a global increase in protein synthesis, which is required for the activation and differentiation of NSCs. Study of ribosomophaties also highlights the importance of the ribosome in differentiation. Ribosomopathies are a group of inherited human diseases that are caused by mutations in the small or large ribosomal subunits or factors involved in ribosome biogenesis (Tahmasebi et al., 2018a). While ribosomes can be found in almost all mammalian cells, it is surprising that defects in ribosomal function preferably affect only specific cell types, most prominently erythroid progenitors. Several hypotheses have been proposed to explain the cell type and tissue specificity associated with ribosomopathies. One model suggests that ribosomes are heterogeneous and each cell type possesses its own unique set of ribosomes, which are specialized in translating cell type-specific mRNAs (specialized ribosome model) (Xue and Barna, 2012; Shi et al., 2017). An alternative model suggests that ribosomes are homogenous, but different mRNAs or cell types have different sensitivity to ribosomal defects (ribosome concentration model) (Mills and Green, 2017). For instance, studies in Diamond-Blackfan anemia (DBA) demonstrated that mutations in 60S or 40S ribosomal proteins [such as RPL5, RPL11, RPS7, RPS10 among others (Tahmasebi et al., 2018a)] decrease the ribosome levels but leave the composition of the ribosomes intact. This renders ribosome availability a limiting factor for translation of a subset of mRNAs, such as GATA1, that play a critical role in differentiation of HSCs (Khajuria et al., 2018). In support of this model, mutation of other factors that impair ribosome biogenesis have been linked to depletion of HSCs (Le Bouteiller et al., 2013).

### mTORC1/4E-BPs

The importance of the mTORC1/4E-BPs pathway in self-renewal and differentiation of stem cells is well documented in ESCs, HSCs, and NSCs (Sampath et al., 2008; Hartman et al., 2013; Signer et al., 2014, 2016; Tahmasebi et al., 2016). ESCs have the remarkable ability to maintain low mTORC1 activity in the presence of LIF (an activator of the PI3K-Akt pathway) and a high content of amino acids and serum (15% FBS) in the medium (Sampath et al., 2008; Tahmasebi et al., 2014, 2016). Combining polysome profiling with microarray analysis, Sampath et al. (2008) discovered a hierarchical translation control network downstream of the mTORC1/4E-BP pathway that regulates expression of pro-differentiation mRNAs. mTOR activity and phosphorylation of 4E-BP1 increase in response to ESC differentiation. The importance of 4E-BPs in the regulation of self-renewal and differentiation of ESCs has been also examined in ESCs lacking 4E-BP1 and 2 (the two 4E-BP isoforms that are highly expressed in ESCs). 4E-BP1/2 DKO ESCs proliferate slower than WT cells and are prone to differentiation partly through increased translation of *YY2* mRNA (Tahmasebi et al., 2016). In addition, the mTORC1/4E-BP pathway plays a critical role in the generation of induced pluripotent stem cells (iPSCs) (Chen et al., 2011; He et al., 2012; Tahmasebi et al., 2014). Interestingly, more recent evidence indicates that ESCs are largely tolerant to mTOR inhibition. Inhibition of the mTOR pathway by mTOR inhibitors (INK128 and RapaLink-1) engenders a reversible paused state in ESCs and blastocysts. Paused ESCs are translationally and transcriptionally silent, but remain pluripotent, mimicking a diapause state of blastocysts *in vivo* (Bulut-Karslioglu et al., 2016).

There is increasing evidence that the mTOR/4E-BP pathway also contributes to translation inhibition and maintenance of adult stem cells such as HSCs and NSCs. Phosphorylation of 4E-BP1 is reduced in HSC/MPPs (multipotent progenitors) compared to most progenitor cells, and abrogation of 4E-BP1 and 2 specifically increases protein synthesis in HSCs, while having a negative effect on their ability for reconstitution (Signer et al., 2016). The subventricular zone (SVZ) in the fetal and adult brain of mammals harbors a small population of cells with stem cell properties (self-renewal and multipotency), known as NSCs (Figure 1E; Gage, 2000). Hartman et al. (2013) demonstrated that the mTORC1 is suppressed in quiescent NSCs located at the SVZ. The activity of mTORC1 is increased (judged by phosphorylation of 4E-BP1/2 and ribosomal protein S6) in proliferating NSC progenitors undergoing differentiation. Genetic (shRNA against Rheb or Raptor) or pharmacological (rapamycin) inhibition of mTORC1 blocks differentiation of NSCs to intermediate progenitors, resulting in lower neuron production. Hyperactivation of mTORC1 mediated by a constitutively active Rheb (Rheb^CA^) induces differentiation of NSC and reduces the population of self-renewing NSCs, specifically through inhibition of 4E-BPs (Hartman et al., 2013).

### ISR Pathway

Recent studies highlighted the importance of the ISR pathway in translational control of stem cells. The ISR pathway activation is triggered by a family of four kinases that control translation initiation through phosphorylation of eIF2α. The eIF2α kinases encompass HRI (heme-regulated inhibitor; also known as eIF2AK1), PKR (protein kinase RNA-activated; also known as eIF2AK2), PERK [PKR-like endoplasmic reticulum (ER) kinase; also known as eIF2AK3], and GCN2 (general control nonderepressible 2; also known as eIF2AK4). All four kinases share a conserved kinase domain but each has evolved unique regulatory domains that only sense and respond to a distinct set of stressors. While p-eIF2α decreases global translation, it has a stimulatory effect on the translation of selective mRNAs containing uORFs within their 5′UTR such as *Atf4*, *Chop*, and *BiP*. By suppressing global translation but increasing translation of stress-induced mRNAs, cells can overcome the stress condition. The significance of HRI and PERK in erythropoiesis and differentiation of pancreatic beta cells, respectively, has been uncovered using transgenic animal models (Han et al., 2001; Harding et al., 2001; Zhang et al., 2006). The discovery of PERK mutations in Wolcott-Rallison syndrome (WRS), a multi-systemic disease with early-onset diabetes mellitus, further supports the findings in animal models (Delepine et al., 2000). Additionally, genome-wide translational profiling underscores the importance of eIF2 phosphorylation in erythroid homeostasis (Paolini et al., 2018). Increasing evidence in recent years emerged that demonstrate the importance of the ISR pathway in stem cells. Zismanov et al. (2016) used the *eIF2α*^S51A/S51A^ mouse model (where phosphorylation of eIF2α has been blocked by mutation of serine 51 to alanine) to highlight the significance of eIF2α phosphorylation in muscle stem cells. Muscle stem cells, also known as satellite cells, are a small population of cells located between sarcolemma and the basal lamina of muscle fibers (Figure 1F), and play a critical role in growth and regeneration of muscles. In quiescent satellite cells, the level of p-eIF2α is high but it quickly decreases once the cells differentiate and start to activate the myogenic program. The high level of p-eIF2α in the quiescent satellite cells has been linked to relatively high activity of PERK in these cells. Zismanov et al. (2016) further showed that in addition to the well-characterized p-eIF2α targets (e.g., Atf4 and Chop), translation of numerous stem cell-related mRNAs such as the deubiquitinating enzyme *Usp9x* (Ivanova et al., 2002; Ramalho-Santos et al., 2002) relies on p-eIF2α. Importantly, a chemical-mediated increase in p-eIF2α (using sal003, a compound that inhibits the eIF2α phosphatase Gadd34/PP1) promotes self-renewal and regenerative capacity of cultured satellite cells, indicating that modulation of p-eIF2α can be used as a strategy to improve stem cell transplantation. The mTORC1 pathway also regulates the activity of satellite cells and is required for their transition from G0 quiescent state into G_Alert_ phase (an “alerting” state of quiescent stem cells that allows them to immediately enter the cell cycle and respond to injury or stress) (Rodgers et al., 2014). Thus, in addition to p-eIF2α, it is highly likely that the activity of 4E-BPs contributes to translation inhibition in satellite cells.

Studies in other stem cell populations also uncovered the importance of p-eIF2α in self-renewal and differentiation. Undifferentiated ESCs have a high level of p-eIF2α, while differentiation decreases p-eIF2α levels (Friend et al., 2015). p-eIF2α promotes translation of stem cell factors, such as *Nanog* and *Myc* containing uORFs in their 5′UTR. A study in human HSCs demonstrated that PERK and PERK-dependent genes (*Atf4*, Chop, and *Gadd34)* are enriched in HSPCs (HSCs and progenitor cells; CD34^+^CD38^-^) as compared to more differentiated progenitors (CD34^+^CD38^+^) (van Galen et al., 2014). Accordingly, HSCs display a higher sensitivity (increased apoptosis and reduced clonogenic capacity) to ER stress compared to progenitors. Overexpression of ERDJ4 (a member of the J protein family that fosters protein folding in ER) in HSCs decreases ER stress and promotes *in vivo* transplantation (van Galen et al., 2014).

### Other Translation Factors

It is very likely that additional translational factors or regulators contribute to translational control in stem cells. For instance, recent data support the importance of m(6)A RNA modification in differentiation of ESCs (Batista et al., 2014). Despite the long list of biochemically characterized eIFs, only few studies examined the role of eIFs in stem cells. Lack of eIFs in mouse is often embryonic or perinatal lethal and has detrimental effects on stem cells and normal development (Table 1).

**Table 1:** Lethal phenotypes resulting from change in activity or lack of eIFs in mouse.

Gene	Lethal phenotype	Reference
Eif2b3	Preweaning or embryonic lethality, complete penetrance, decreased hemoglobin content	Meehan et al., 2017
Eif2b4	Preweaning lethality, complete penetrance, enlarged heart	Meehan et al., 2017
eIF2alpha^S51A^	Neonatal lethality, complete penetrance, neonates died within 18 h after birth	Scheuner et al., 2001
Ppp1r15b	Preweaning lethality, all die in the first day of postnatal life	Harding et al., 2009
Ppp1r15a; Ppp1r15b	Embryonic lethal, Embryo die before preimplantation period	Harding et al., 2009
Eif4e	Embryonic lethal, Embryo die before E6.5	Truitt et al., 2015
Eif4e2	Perinatal lethality	Morita et al., 2012
Nat1/Eif4G2	Embryonic lethal, defects in gastrulation	Yamanaka et al., 2000
eIF3m	Embryonic lethal at the peri-implantation stage	Zeng et al., 2013
eIF3e	Embryonic lethal, Embryo die before E10.5	Sadato et al., 2018
Dhx29	Preweaning lethality, complete penetrance	International Mouse Phenotyping Consortium (IMPC)


### DAP5/p97/NAT1, eIF4G2

Nat1 (also known as DAP5 and eIF4G2) is an eIF4G homolog that interacts with eIF4A, eIF3, and MNK. However, in contrast to eIF4G, p97/DAP5/Nat1 does not bind to eIF4E and therefore has been proposed to be involved in cap-independent translation (Henis-Korenblit et al., 2002; Liberman et al., 2015). Nat1 KO mice are embryonic lethal and display defects in the gastrulation step (Yamanaka et al., 2000). Proliferation and global translation are similar between Nat1 null ESCs and their WT counterpart. However, Nat1 null cells are resistant to differentiation in both mouse and human (Yamanaka et al., 2000; Yoffe et al., 2016). Ribosome foot-printing analysis of Nat1 KO ESCs demonstrated that lack of Nat1 causes a decrease translation of differentiation-promoting factors such as Map3k3 and Sos1 (Sugiyama et al., 2017).

## Translation Inhibition in Stem Cells is Cell Cycle Independent

Studies on embryonic and adult stem cells demonstrated that translation inhibition is independent of replication rate in these cells. Mouse ESCs exhibit a fast replication rate (divide every 8–10 h as compared to >16 h of differentiated cells), and have a unique cell cycle control (Singh and Dalton, 2009), as they progress through a very short G1 phase (15%), while residing mostly in S phase (65%). Human ESCs maintain similar cell cycle structure as mouse ESCs, however, they replicate much slower (divide every 30–38 h) (Singh and Dalton, 2009). Adult stem cells are slow-growing cells that spend most of their time in a dormant state (G0/G1) and only divide in response to physiological or pathological stimuli. Low translation rate of HSCs is not just a consequence of their dormant state, as when protein synthesis was compared using cell cycle-matched populations (S/G2/M or G0/G1), HSCs exhibited a lower translation rate compared to differentiated progenitors (Signer et al., 2014). Study in HFSCs also demonstrates that the rate of protein synthesis is independent of cell cycle and proliferation (Blanco et al., 2016). How stem cells decouple translation rate from cell cycle control has yet to be understood, and remains one of many intriguing questions in the stem cell field.

## Concluding Remarks

It has been more than five decades since the importance of translation control in early developmental processes was delineated through the study of the fertilization of sea urchin eggs (Hultin, 1961; Monroy et al., 1961; Tahmasebi et al., 2018b). However, the role of translational control in differentiation and maintenance of stem cells has been explored only recently. Technological advances in the studies of translation, combined with novel genetic approaches, are beginning to provide the essential tools required for understanding this critical step of gene expression in stem cell plasticity.

## Author Contributions

All authors contributed in conceptualizing and writing the review.

## Conflict of Interest Statement

The authors declare that the research was conducted in the absence of any commercial or financial relationships that could be construed as a potential conflict of interest.
